# Expression of *Pseudomonas syringae* type III effectors in yeast under stress conditions reveals that HopX1 attenuates activation of the high osmolarity glycerol MAP kinase pathway

**DOI:** 10.1099/mic.0.X00007-0

**Published:** 2013-01

**Authors:** Dor Salomon, Eran Bosis, Daniel Dar, Iftach Nachman, Guido Sessa

The data in Table 1 of the above paper are incorrect. A corrected version of the table is given below.

**Table 1.** Yeast growth inhibition phenotypes induced by *Pst* T3Es under various stress conditions BY4741 yeast strains carrying plasmids for the expression of the indicated effectors or containing an empty vector were grown overnight in repressing medium (2 % glucose). Cultures were then washed, normalized to OD_600_ 1.0, and 10-fold serial dilutions were spotted onto repressing medium or onto inducing medium (2 % galactose and 1 % raffinose), and grown under the following stress conditions: in the presence of 7 mM caffeine, 0.5 M sodium chloride (NaCl), 1 M sorbitol, 0.12 µg ml^−1^ tunicamycin (Tm), or at a temperature of 37 °C. None: growth under normal conditions without stress. Two to four days after spotting, the highest dilutions in which colony growth was visible were determined for each strain, and reported according to the growth scale below. Data shown are representative of three independent experiments for each effector. Degree of growth: −−−−, no growth in any dilution; −−−, up to the 10^0^ dilution; −−, up to the 10^−1^ dilution; −, up to the 10^−2^ dilution; no sign, up to the 10^−3^ dilution. Please note that for the effectors AvrPto, HopA1, HopAA1-2*, HopAF1, HopB1, HopC1, HopE1, HopF2, HopI1, HopK1, HopM1*, HopN1, HopO1-1, HopQ1, HopV1, HopY1 and HopR1* the degree of growth was up to the 10^−3^ dilution under all conditions (not shown).

Effector	None	Caffeine	NaCl	Sorbitol	Tm	37 °C
Empty						
HopAA1-1*	−−−−	−−−−	−−−−	−−−−	−−−−	−−−−
HopAM1	−−−−	−−−−	−−−−	−−−−	−−−−	−−−−
HopU1	−−−−	−−−−	−−−−	−−−−	−−−−	−−−−
AvrE1*	−−−−	−−−−	−−−−	−−−−	−−−−	−−−−
HopAD1*	−−−−	−−−−	−−−−	−−−−	−−−−	
HopAO1	−	−−	−−−−	−−−−	−	−−−−
HopD1	−	−	−−	−−	−−	−−
HopX1		−	−−−−	−−−−		−
HopG1			−−−	−		−−
HopT1-1			−−−	−		
HopH1		−	−−			
AvrPtoB					−	

* Indicates effectors whose expression was not detected in immunoblot analysis.

doi:10.1099/mic.0.X00007-0

